# Extravasation injury management for neonates and children: A systematic review and aggregated case series

**DOI:** 10.1002/jhm.12951

**Published:** 2022-08-30

**Authors:** Mitchell Dufficy, Mari Takashima, Jacqueline Cunninghame, Bronwyn R. Griffin, Craig A. McBride, Deanne August, Amanda J. Ullman

**Affiliations:** ^1^ School of Nursing, Midwifery and Social Work The University of Queensland Brisbane Queensland Australia; ^2^ Centre for Children's Health Research Children's Health Queensland Hospital and Health Service Brisbane Queensland Australia; ^3^ School of Nursing and Midwifery Griffith University Brisbane Queensland Australia; ^4^ Grantley Stable Neonatal Unit Royal Brisbane and Women's Hospital Brisbane Queensland Australia

## Abstract

**Background:**

Pediatric extravasation injuries are significant healthcare‐associated injuries, with sometimes significant sequelae. Evidence‐based guidance on management is necessary to prevent permanent injury.

**Purpose:**

A systematic review of the literature, including aggregated case series, investigating extravasation injury management of hospitalized pediatric patients.

**Data Sources:**

PubMed, Cummulative Index to Nursing and Allied Health Literature (CINAHL), and Excerpta Medica database (EMBASE) were searched on December 13, 2021.

**Study Selection:**

Primary research investigating extravasation injury management of hospitalized pediatric patients (to 18 years), published from 2010 onwards and in English, independently screened by two authors, with arbitration from a third author.

**Data Extraction:**

Data regarding the study, patient (age, primary diagnosis), extravasation (site, presentation, outcome), and treatment (first aid, wound management) were extracted by two authors, with arbitration from a third author.

**Data Synthesis:**

From an initial 1769 articles, 27 studies were included with extractable case data reported in 18 studies, resulting in 33 cases. No clinical trials were identified, instead, studies were primarily case studies (52%) of neonates (67%), with varied extravasation symptoms. Studies had good selection and ascertainment, but few met the causality and reporting requirements for quality assessments. Signs and symptoms varied, with scarring (45%) and necrosis (30%) commonly described. Diverse treatments were categorized into first aid, medical, surgical, and dressings.

**Conclusions:**

Despite infiltration and extravasation injuries being common within pediatric healthcare, management interventions are under‐researched, with low‐quality studies and no consensus on treatments or outcomes.

## INTRODUCTION

Access to the peripheral venous system for the administration of fluids and medications is a fundamental procedure within healthcare, particularly among children.^1^, ^2^ However, the use of peripheral intravenous catheters (PIVCs) is not without risk of harm. Extravasation injuries are caused by the inadvertent administration of vesicant and irritant solutions into the tissues surrounding the PIVC vessel.^3^

Young children (including neonates) and those with conditions affecting communication are especially vulnerable to significant extravasation injuries due to their inability to report pain, their skin and vein fragility, and carer and staff difficulties inspecting insertion sites.^4^, ^5^ Up to 11% of pediatric patients and 70% of neonates receiving intravenous therapy will experience extravasation of an intravenous infusion.^4^, ^6^, ^7^, ^8^, ^9^ The significance of such an injury varies and is highly dependent on the location, the medication or fluid involved, concentration and volume of extravasate, dilutant used for reconstitution, reaction site, condition of surrounding skin, and time of detection and treatment.^1^, ^3^ The trauma caused by such injuries can progress to scarring and/or surgical excision of the affected area, skin grafting, and functional loss.^3^

Approaches to managing extravasation primarily concentrate on pharmacological and nonpharmacological interventions to aid in preventing tissue damage, reversal agents specific to the type of extravasation, and surgical intervention if necessary.^10^ The relative effectiveness of these strategies, across the variety of extravasation injuries that present in pediatrics is unclear. The aim of this study is to describe and evaluate current extravasation injury management and treatment options in use for children with extravasation, with the aim of reducing scarring and necrosis of tissue.

## METHODS

### Design

A systematic review including synthesis via aggregated case series has been undertaken to answer the following research question and outcomes:

1. What extravasation injury management strategies are in use for hospitalized children?
2. How effective are these approaches to reduce tissue necrosis and scarring of the extravasation injury area, symptoms (pain, stinging, burning, edema around the injection site), tissue dysfunction, physical disability, compartment syndrome, and to improve quality of life?

Case reports and case series have been part of the importance of healthcare research to alert and educate fellow clinicians when robust evidence is not available.^11^ Most case reports and series have been treated as a single entity without being aggregated,^11^ despite their detailed description of the sequence and management of patient care. Aggregated case series methods proposed by Murad^12^ suggest using evidence derived from case reports and case series to inform decision‐making when no higher level evidence is available. The tool also evaluates the methodological quality in the domains of selection, ascertainment, causality, and reporting to aid the clinicians with the quality assessment of the included study. Additionally, aggregated case series also make subgroup comparisons (e.g., age, diagnosis, gender) to allow for subgroup‐specific differences to be identified, such as in signs and symptoms and case management, for example.

The review was prospectively registered with PROSPERO CRD42022307906 and has been reported in accordance with Preferred Reporting Items for Systematic Reviews and Meta‐Analyses (PRISMA) guidelines for systematic reviews.^13^

### Search strategy and information sources

With the aid of the health librarian, search terms were developed utilizing medical subject headings (MeSH) of “Extravasation of Diagnostic and Therapeutic Materials,” “Child, Hospitalized,” and “necrosis” and other correlating search terms associated with the patient/population, intervention/indicator, compare/control, and outcome (PICO) formatted research question. The search was performed on December 13, 2021 and accessed the following databases: PubMed, Cumulative Index to Nursing and Allied Health Literature (CINAHL), and Excerpta Medica database (EMBASE). Supporting Information: Table 1 depicts the search strategy used for PubMed in this systematic review.

### Eligibility criteria

Exclusion and inclusion criteria are outlined in Table 1 and were used to scrutinize search results from the search strategy. Furthermore, restrictions of studies published in English and a date limit of 2010 onwards were applied, to ensure contemporaneous surrounding practices. The reference lists of included articles were searched individually for other relevant articles to identify further literature outside of the search strategy that is applicable to the research questions.

**Table 1:** Inclusion and exclusion criteria

	Inclusion	Exclusion
Date range	Publications 2010 onwards	Publications older than 2010
Paper type	Randomized controlled trials Cohort studies (including pre‐post) Case‐controlled studies Case study/case series	Background information/expert opinion Systematic review Critically appraised topics (evidence syntheses and guidelines) Critically appraised individual articles (article synopses)
Language	English language	Non‐English language
Population	Children aged under 18 admitted to the hospital with a peripheral intravenous catheter that extravasated	Over 18 years of age
Full‐text	Publications with a full‐text	No full‐text

### Study selection

Duplications were removed and titles and abstracts were screened against the inclusion and exclusion criteria independently by two authors (M. D. with J. C. or A. J. U.) using Covidence®.^14^ Full‐text review was performed independently by the same process. Where eligibility was unclear arbitration was sought from the senior author. Full text of all articles included in the full‐text review was sought through an institutional loan; however, a small number of articles that were unable to be obtained were excluded, and the first authors of the articles were not contacted.

### Data extraction and quality appraisal

Data were extracted and reviewed for quality from eligible articles using Covidence. Reviews were independent by the first author (M. D.) followed by a secondary author (J. C.), with arbitration sought from a third independent author (A. J. U.) when a consensus was not achieved. A standardized data extraction form included the author, year, journal name, title, country of origin, study design, population type, primary diagnostic group, and control. For articles reporting individual case data (demographics), signs and symptoms, treatment pathway, management, and outcome (wound healing, side effects, and days taken to wound to heal), the data were extracted by the second author (M. T.) and checked by the first author (M. D.). Before use, these forms were checked by clinical experts in pediatrics, intravenous catheters, and wound care. Cases were only included once.

Quality assessment was achieved by the Murad^12^ synthesis scale to assess the article against the selection, ascertainment, causality, and reporting criteria. This process was led by the first author (M. D.) in conjunction with three additional authors (A. J. U., M. T., or J. C.).

### Data synthesis

Data were exported from Covidence into a Microsoft Excel® spreadsheet by the first author (M. D.). Study designs were categorized according to published definitions.^15^ Narrative synthesis was undertaken by another author (M. T.) to ascertain what aspects of the literature could be developed and represented in tables to effectively answer the review questions. The data were then reviewed, categorized, and displayed in relevant tables and graphs.

### Synthesis of case data

Quantitative case synthesis of individual case data was undertaken and reported by categorizing into age subgroups: neonates (<1 month old), children (1 month old to 10 years old), and adolescents (greater than 10 years old).^12^ Results are descriptively reported.

## RESULTS

### Study selection

**Figure 1:**
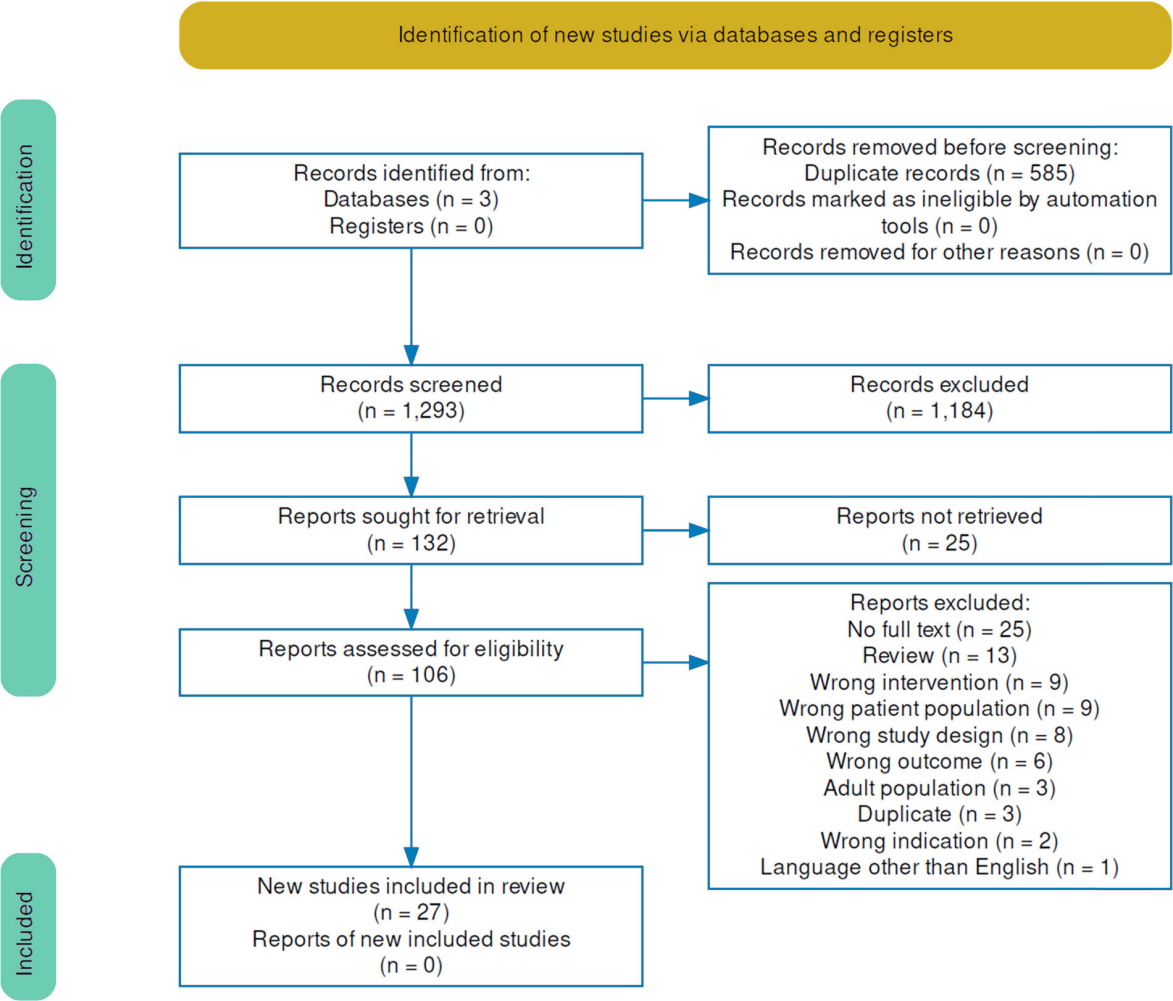
Preferred Reporting Items for Systematic Reviews and Meta‐Analyses systematic review flow chart.

Figure 1 shows the study selection in accordance with the PRISMA recommendations. The search yielded an initial result of 1769 articles, a collective result of the selected databases. Once duplications were removed, 1184 papers' titles and abstracts were screened, resulting in 79 studies. The full texts of these 79 studies were assessed against the inclusion criteria, resulting in 27 studies to extract for the final analysis. Extractable case data were reported in 18 studies resulting in 33 cases, which mainly came from case studies.

### Study characteristics

Individual characteristics of included studies (*n* = 27) are depicted in Table 2. Included studies were published between 2010 and 2021, predominantly within the United States (*n* = 8), and primarily included case series.

**Table 2:** Characteristics of included studies (*N* = 27 studies)

Characteristics	*N* (%)
*Year of publication*	
2021	4 (15)
2019	3 (11)
2018	5 (19)
2017	2 (7)
2016	1 (4)
2015	4 (15)
2014	3 (11)
2012	2 (7)
2011	1 (4)
2010	2 (7)
*Country of origin*	
USA	8 (30)
UK, France, Germany, Italy	7 (26)
Korea, Taiwan, China	5 (19)
Iran, Turkey, Greece	4 (15)
Australia	1 (4)
Peru	1 (4)
N/A	1 (4)
*Study type*	
Case series	14 (52)
Case study	9 (33)
Prospective cohort	1 (4)
Retrospective cohort	2 (7)
Prospective study/case series	1 (4)
*Age group*	
Neonates	18 (67)
Children	3 (11)
Adolescents	1 (4)
Mixed	5 (19)
*IV medication extravasated*	
Fluids and electrolytes	7 (26)
Parenteral nutrition	6 (22)
Antibiotics	2 (7)
Cytotoxic	2 (7)
Mixed	6 (22)
Not reported	4 (15)

### Critical appraisal and quality assessment

Included publications were predominantly case reports and series (see Supporting Information: Table 1). The article's quality assessments had a good selection and ascertainment; however, a few of the studies did not meet the requirements for causality and reporting.

### Findings

#### Demographics

Among extracted cases, males represented more than half of the neonates (*n* = 10; 56%), adolescents (*n* = 2; 67%), and overall (*n* = 17; 52%). Primary diagnosis was mostly prematurity (*n* = 7; 41%) and respiratory (*n* = 6; 35%) for neonates; respiratory distress (*n* = 3; 33%) for children; and surgery and trauma (1; 50% each) for adolescents. About 60% (*n* = 10) of PIVCs were inserted in the foot/leg for neonates and 1/3 (*n* = 4) for children. Around 60% of the medication that caused extravasation was parenteral nutrition for neonates, whereas half of the medications were fluids and electrolytes for children and adolescents.

#### Treatment types

The treatment types were extracted and reported at the study level. There were no differences between treatment types among age groups from aggregated case series.

#### First aid

**Figure 2:**
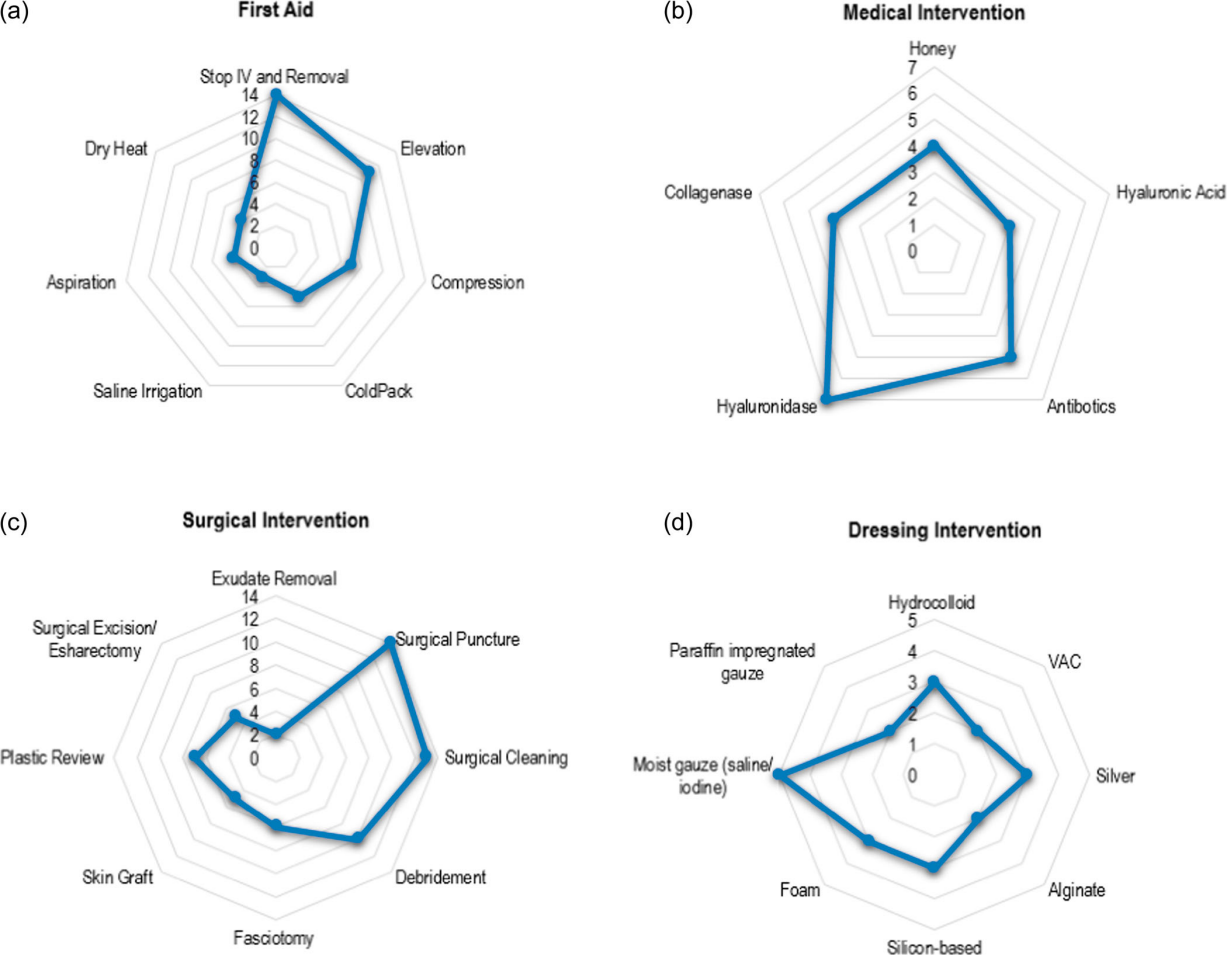
Interventions* (*N* = 27 studies). The numbers shown in the middle of the radar chart are the total number of each intervention utilized within the included studies, with each intervention displaying a dot on the corresponding accumulation number line. Interventions not displayed in the chart were not utilized as often. *The interventions that only had one count (otherwise described): (a) First aid: Duoderm gel, hydrocolloid dressing, physical therapy, 5% povidone‐iodine, pulse oximetry, burns service team, massage, scrub with saline wetted gauze; (b) Medical: hydrolyzed collagen powder, oxacillin, silver nitrate, analgesia, phenotolamine, topical oil moisturization, sodium hypochlorite with saline, topical nitroglycerine ointment, allopurinol treatment, fusidic acid, dermatix silicone gel, and 10% sodium chloride; (c) Surgical: open amputation; (d) Dressing: hydrofiber, collagen, surgical tape strips, nonadherent compressive, Mepilex® (3; but not detailed).

First aid interventions were primarily used in conjunction with medical, surgical, and dressing interventions. First aid interventions, as shown in Figure 2a, were identified in 26 studies.^16^, ^17^, ^18^, ^19^, ^20^, ^21^, ^22^, ^23^, ^24^, ^25^, ^26^, ^27^, ^28^, ^29^, ^30^, ^31^, ^32^, ^33^, ^34^, ^35^, ^36^ Stopping the IV and removal was the most common intervention,^16^, ^17^, ^18^, ^19^, ^22^, ^23^, ^25^, ^26^, ^28^, ^29^, ^30^, ^31^, ^34^, ^35^ followed by elevation^16^, ^17^, ^19^, ^20^, ^21^, ^24^, ^25^, ^26^, ^28^, ^29^, ^31^, ^32^ and compression.^17^, ^27^, ^29^, ^31^, ^32^, ^33^, ^36^

#### Medical

Medical interventions, as shown in Figure 2b, were identified in 18 studies, the most common being hyaluronidase,^18^, ^21^, ^29^, ^31^, ^35^, ^36^, ^37^ with some studies using it in conjunction with the wash‐out technique to increase positive outcomes. Antibiotics,^19^, ^24^, ^26^, ^27^, ^37^ honey,^34^, ^37^, ^38^, ^39^ collagenase,^24^, ^30^, ^37^, ^38^ and hyaluronic acid^18^ were also utilized.

#### Surgical

Surgical interventions, as shown in Figure 2c, were utilized in 22 studies.^16^, ^17^, ^19^, ^20^, ^21^, ^22^, ^23^, ^24^, ^25^, ^27^, ^28^, ^30^, ^31^, ^32^, ^33^, ^34^, ^35^, ^36^, ^37^, ^38^, ^40^, ^41^, ^42^ Surgical puncture^17^, ^21^, ^22^, ^23^, ^25^, ^28^, ^30^, ^31^, ^33^, ^35^, ^37^, ^40^, ^41^, ^42^ and surgical cleaning^17^, ^21^, ^23^, ^25^, ^28^, ^30^, ^31^, ^32^, ^33^, ^35^, ^37^, ^40^, ^41^ were most routinely used among the studies. Moreover, debridement,^16^, ^17^, ^23^, ^24^, ^30^, ^33^, ^34^, ^37^, ^38^, ^41^ fasciotomy,^19^, ^25^, ^32^, ^34^, ^40^, ^42^ surgical excision/escharectomy, plastic surgical review,^16^, ^19^, ^21^, ^23^, ^28^, ^31^ and skin graft^20^, ^22^, ^23^, ^24^, ^40^ were also utilized. There were seven traditional/surgical debridements,^16^, ^23^, ^24^, ^30^, ^34^, ^37^, ^41^ three enzymatic debridements,^37^, ^38^, ^41^ three autolytic debridements,^17^, ^37^, ^41^ two mechanical debridements,^37^, ^38^ and one Versajet™ hydrosurgery system debridement.^33^

#### Dressings

The use of dressings as an intervention, as shown in Figure 2d, was evident within 21 studies.^16^, ^17^, ^19^, ^22^, ^23^, ^24^, ^25^, ^27^, ^28^, ^29^, ^30^, ^31^, ^32^, ^33^, ^34^, ^36^, ^37^, ^38^, ^40^, ^41^, ^42^ Moist gauze (saline/iodine) was the most used dressing, followed by foam, silicone‐based, silver, and hydrocolloid dressings. Dressings used throughout the studies were primarily used after the extravasation injury had been managed with alternative interventions. For example, in Ahmadli's^16^ study, first aid measures were undertaken upon identifying the extravasation, such as removing the PIVC, applying a cold pack, elevating the site, and cleaning the site. Debridement then occurred, and multiple types of dressings were applied to subsequently manage the wound.

#### Primary and secondary outcomes

The definition of wound healing varied between studies. Accurate data extraction and collation from the case studies/series was challenging. For example, Murphy et al.'s^28^ study recorded the time of wound healing of one participant as 150 days whereas Odom et al.'s^29^ study recorded an average time of wound healing as 19 days. This vast variation in wound healing time is applicable to the absent consented definition as some may perceive this term as simple wound healing in comparison to a full recovery with function. Likewise, the absence of a unanimous measuring tool to assess the extravasation injury, such as ultrasound or visual assessment, results in the heterogenous classification of the wound healing stage.

In aggregated case series, neonatal cases reported a median of 34 days (interquartile range [IQR]: 21–60) for the wound to heal; 25 (IQR: 7–98) days for children; and 276 (IQR: 13–540) days for adolescents. The definition of healed wounds was author‐defined, hence the varied and wide range of days taken for wound healing.

#### Extravasation sequelae

As shown in Table 3, 21 studies identified what was caused by the extravasation prior to treatment,^16^, ^17^, ^18^, ^19^, ^20^, ^21^, ^22^, ^23^, ^25^, ^26^, ^31^, ^32^, ^33^, ^34^, ^35^, ^36^, ^37^, ^39^, ^40^, ^41^, ^42^ which most commonly included swelling,^17^, ^18^, ^19^, ^26^, ^33^, ^36^, ^40^, ^41^, ^42^ blisters,^16^, ^17^, ^20^, ^25^, ^33^, ^37^, ^40^, ^42^ pain,^16^, ^23^, ^26^, ^36^, ^42^ skin necrosis,^16^, ^20^, ^21^, ^22^, ^25^, ^32^, ^33^, ^34^, ^35^, ^36^, ^37^, ^41^ edema,^16^, ^17^, ^20^, ^22^, ^25^, ^32^, ^34^, ^37^, ^39^, ^42^ and altered skin color/blanching.^17^, ^23^, ^25^, ^31^, ^33^, ^40^, ^41^, ^42^

**Table 3:** Characteristics of extracted cases of extravasation by age group (*N* = 33)

Feature	Neonates (<1 month) (%) (*N* = 18)	Child (1 month to 10 years old) (%) (*N* = 12)	Adolescent (≥10 years old) (%) (*N* = 3)	Total (%) (*N* = 33)
*Sex category*				
Female	5 (28)	1 (8)	0 (0)	6 (18)
Male	10 (56)	5 (42)	2 (67)	17 (52)
Unknown	3 (16)	6 (50)	1 (33)	10 (30)
*Primary diagnosis*				
Cancer	0 (0)	1 (11)	0 (0)	1 (4)
Congenital disease	4 (24)	2 (22)	0 (0)	6 (21)
Dehydration	0 (0)	1 (11)	0 (0)	1 (4)
Prematurity	7 (41)	1 (11)	0 (0)	8 (28)
Respiratory distress	6 (35)	3 (33)	0 (0)	9 (31)
Sepsis	0 (0)	1 (11)	0 (0)	1 (4)
Surgery	0 (0)	0 (0)	1 (50)	1 (4)
Trauma	0 (0)	0 (0)	1 (50)	1 (4)
*IV location*				
Foot/leg	10 (59)	4 (33)	0 (0)	14 (44)
Hand/arm	7 (41)	8 (67)	3 (100)	18 (56)
*Medication that caused extravasation*				
Antibiotics	0 (0)	2 (17)	0 (0)	2 (6)
Cytotoxic	0 (0)	1 (8)	1 (33)	2 (6)
Fluids and electrolytes	3 (17)	6 (50)	2 (67)	11 (33)
Parenteral nutrition	10 (56)	2 (17)	0 (0)	12 (37)
Mixed	2 (10)	1 (8)	0 (0)	3 (9)
Not reported	3 (17)	0 (0)	0 (0)	3 (9)
*Signs and symptoms before treatment* a				
Edema/swollen site	9 (50)	8 (67)	0 (0)	17 (52)
Discoloration	1 (6)	3 (25)	0 (0)	4 (13)
Necrosis	5 (28)	2 (17)	0 (0)	7 (21)
Blisters	2 (12)	3 (25)	0 (0)	5 (16)
Diagnosis of extravasation	3 (17)	3 (25)	1 (33)	7 (21)
Pain/swelling/inflammation/discomfort	1 (6)	5 (42)	0 (0)	6 (18)
Exudates	4 (22)	0 (0)	0 (0)	4 (22)
Compartment syndrome	2 (11)	4 (33)	1 (33)	7 (21)
Other	8 (44)	4 (33)	2 (67)	14 (42)
*Signs and symptoms after the treatment were initiated* a				
Edema/swollen site	2 (11)	1 (8)	0 (0)	3 (9)
Scarring	7 (39)	1 (8)	0 (0)	8 (24)
Necrosis	2 (11)	2 (17)	1 (33)	5 (15)
Calcinosis cutis	1 (6)	0 (0)	0 (0)	1 (3)
Pain/swelling/inflammation/discomfort	0 (0)	3 (25)	0 (0)	3 (9)
Compartment syndrome	0 (0)	1 (17)	0 (0)	1 (4)
Other	3 (17)	5 (42)	1 (33)	9 (27)
*Signs and symptoms resolved addressed from treatment* a				
Edema/swollen site	3 (17)	0 (0)	0 (0)	3 (9)
Exudate	2 (11)	0 (0)	0 (0)	2 (6)
Tissue dysfunction/physical defects	7 (39)	6 (26)	0 (0)	13 (39)
Compartment syndrome	0 (0)	2 (9)	2 (67)	2 (6)
Pain/swelling/inflammation/discomfort	2 (11)	2 (9)	0 (0)	4 (12)
Healed wound	4 (22)	2 (9)	0 (0)	6 (18)
Discoloration	2 (11)	0 (0)	0 (0)	0 (0)
Necrosis	0 (0)	1 (4)	0 (0)	1 (3)
Not well reported	6 (33)	5 (22)	1 (33)	14 (42)
*Long‐term side effects*				
Keloid scars	0 (0)	1 (4)	0 (0)	1 (3)
Retraction; restriction of movements	2 (11)	0 (0)	1 (33)	3 (9)
*Days taken for the wound to heal* b				
Median (interquartile range)	34 (21–60)	25 (7–98) (1 missing)	276 (13–540) (1 missing)	30 (14–67)

^a^ Can have more than one sign and symptom and not all the studies have reported on the same measures.

^b^ The definition of wound healing varies between studies as they each define the term individually, with no consensus made on the definition

In aggregated case series, edema/swelling was the most reported sign and symptom in neonates and children pretreatment (*N* = 23). There were more reports on progressing signs and symptoms after the treatment was initiated across the age groups, for example, scarring, necrosis, and calcinosis cutis. The long‐term side effects were reported as retraction/restriction of movements for neonates and adolescents, and keloid scars for children. The improvement of treatments was often not reported in detail; however, tissue dysfunction and physical defects were the second most reported in neonates and children. Lastly, one study identified slough formation,^37^ agitation/movement restriction,^22^ and identified anemia^32^ as side effects caused by the treatment.

## DISCUSSION

This systematic review effectively describes the documented extravasation injury management strategies and categorizes them in the treatment types section. Additionally, the clinical effectiveness of these approaches to reduce harm are addressed in the primary and secondary outcomes section of this paper. However, in seeking these answers the review found deficits in the extravasation injury management strategies that will be further discussed in the following discussion section.

This systematic review demonstrates that extravasation management is under‐researched in pediatrics. Most studies included neonatal populations only and all management descriptions were observational, and, other than basic first aid, discordant. There were no clinical trials of extravasation management approaches identified; including first aid, medication, surgical, or dressing interventions. This is despite PIVC‐associated extravasation injuries being one of the top quality indicators for pediatric healthcare, with significant injuries estimated to impact ~0.7 children per 1000 patient days, and less significant injuries affecting up to 11% of pediatric patients and 70% of neonates.^4^, ^6^, ^7^, ^43^ The Australian Commission on Safety and Quality in Health Care provides the National Safety and Quality Health Service (NSQHS) Standards a nationally consistent statement on the level of care consumers can expect from health service organizations.^44^ The Medication Safety Standard specifically outlines the health service organization's role in describing, implementing, and monitoring systems to reduce the occurrence of medication incidents, such as extravasation injuries.^45^ For a significant and relatively common healthcare‐associated injury, this dearth of an evidence base supporting practice neither meets the NSQHS Standards nor consumer expectations; the need for the establishment of an evidence base for the management of these injuries is urgent and needed.

Most reported cases of extravasation were parenteral nutrition, hydration fluids, and electrolytes. These are common, necessary infusates, used throughout healthcare. Most infusates documented are not considered “high risk” vesicant solutions, based on existing guidelines.^46^, ^47^ Innovative multimodal methods to prevent, identify, and treat extravasation injuries caused by common infusates must be developed, robustly evaluated, and sustainably implemented.

The identification, diagnosis, and escalation of extravasation are challenging as there are no validated standard diagnostic criteria for extravasation tailored for children, especially when patients are unable to verbalize their pain and discomfort. There have been rudimentary diagnostic tools developed; however, the validity, reliability, and feasibility of these tools are unclear. Within this systematic review, prior diagnostic tools were not routinely used to report all presentations, which made the synthesis of outcomes challenging. Millam's staging^48^ was most widely used within this review, with six studies mentioning the use of Millam's staging; however, only two reported the stages of extravasation of each case^22^, ^25^ whereas the other seven studies used unidentified extravasation diagnostic tools.^18^, ^29^, ^35^, ^36^, ^37^, ^38^, ^39^ There is a need for a universal pediatric‐specific diagnostic tool to be used systematically across hospitals to facilitate benchmarking and collaborative research.

The lack of consistent outcome definitions for wound healing is evident. Due to variable reporting procedures, it was difficult to know if “healing” represented time to spontaneous re‐epithelialization, skin grafting, or time to function improvement. The risk of hypertrophic scarring increases each day, whereby burns that take longer than 3 weeks to heal have higher rates of hypertrophic scarring.^49^ Thereby, the establishment of a unanimous outcome definition to measure the efficacy of treatments and the establishment of unanimous wound healing definitions is pertinent, with the lack of homogeneity in outcome reporting seen in various facets of burn care.^50^ Complications from extravasation injuries can be lifelong and include scarring, leading to contractures and disfigurement, functional loss, complex regional pain syndrome, and deformities.^28^ Other long‐term impacts include eschar formation, tissue ulceration, cosmetic defects, chronic pain, loss of function secondary to contractures or neuropathy, and ulceration of the skin.^51^ With contractures and hypertrophic/keloid scars, children may require repeated scar revisions as they grow to improve their quality of life. Therefore, to identify ideal outcomes of such treatments, consumer engagement in research is recommended and necessary to advise clinicians of their expectations. This knowledge will enable clinicians to target their treatment regime to meet consumer treatment expectations.

This review has significant limitations. Due to limited research encompassing extravasation injury management among the hospitalized pediatric population, this systematic review predominantly utilized case studies as the primary means of data extraction. Additionally, this study utilized publications in English only, meaning some studies may have been excluded. The most important limitation of this systematic review is the inclusion of low‐quality studies due to the inclusion of case reports. The nature of this study design may have a high risk of selection bias and reporting bias. Additionally, since the terminologies are usually author‐defined, we could not systematically and rigorously assess the exposure and outcome. However, we have taken steps to assess the potential risk of bias by implementing the quality assessment.

## CONCLUSION

Ongoing research is necessary to identify and assess the effectiveness of extravasation injury management interventions in pediatrics. Extravasations are one of the most common iatrogenic injuries inflicted on children. They can lead to lifelong sequelae such as scarring, in addition to the pain and suffering caused at the time of injury. Scant evidence is available to support the strategies aimed at treating, preventing, or defining these injuries. Similarly, without a consensus on extravasation injury staging, clinicians may have difficulty recognizing healing and therefore managing injuries appropriately.

## CONFLICT OF INTEREST

The authors declare no conflict of interest.

## ETHICS STATEMENT

The review was prospectively registered with PROSPERO 2022 CRD42022307906. It is available from https://www.crd.york.ac.uk/prospero/display_record.php?ID=CRD42022307906

## Supporting information

Supporting information.
