# Supplementary material for: Extravasation injury management for neonates and children: A systematic review and aggregated case series
Source: J Hosp Med. 2022 Aug 30;17(10):832–42. doi: 10.1002/jhm.12951 (PMC9804918; doi:10.1002/jhm.12951)
Supplement: Supplementary file 1 — Supporting information. [file JHM-17-832-s001.docx]

Supplementary Table 1: Key Characteristics of included articles (n= 27)

| **Author, Citation, Year [ID*]** | **Country** | **Design** | **Age group** | **Extracted**  **Cases^2^** | **Quality assessment^3^** | | | | | | | |
| --- | --- | --- | --- | --- | --- | --- | --- | --- | --- | --- | --- | --- |
|  |  |  |  |  | **Selection** | **Ascertainment** | | **Causality** | | | | **Reporting** |
|  |  |  |  |  | **1** | **2** | **3** | **4** | **5** | **6** | **7** | **8** |
| Ahmadli (2019) | Iran | Case study | Neonates | 1 | **Y** | **Y** | **Y** | **N** | **N** | **N** | **NA** | **Y** |
| Ai-hua (2018) | Unknown | Case series | Neonates | 1 | **Y** | **Y** | **Y** | **N** | **N** | **N** | **NA** | **Y** |
| Boyar (2021) | USA | Case series | Neonates | 2 | **Y** | **Y** | **Y** | **N** | **N** | **N** | **NA** | **Y** |
| Boyar (2018) | USA | Case series | Neonates | 3 | **Y** | **Y** | **Y** | **N** | **N** | **N** | **Y** | **Y** |
| Boyar (2018) | USA | Case series | Neonates | 0 | **Y** | **Y** | **Y** | **N** | **N** | **N** | **NA** | **Y** |
| Boyar (2014) | USA | Case series | Neonates | 1 | **Y** | **Y** | **Y** | **N** | **N** | **N** | **Y** | **Y** |
| Chen (2010) | Taiwan | Case study | Neonates | 1 | **Y** | **Y** | **Y** | **N** | **N** | **N** | **Y** | **Y** |
| D’Acunto (2015) | Italy | Case study | Neonates | 1 | **Y** | **Y** | **Y** | **N** | **N** | **N** | **Y** | **Y** |
| Ghanem (2015) | UK | Prospective / Case series | Mixed | 0 | **Y** | **Y** | **Y** | **N** | **N** | **N** | **N** | **N** |
| Girard (2019) | France | Case series | Neonate/adolescent | 2 | **Y** | **Y** | **Y** | **N** | **N** | **N** | **Y** | **Y** |
| Harb (2010) | UK | Case study | Child | 1 | **Y** | **Y** | **Y** | **N** | **N** | **N** | **NA** | **Y** |
| Hirsch (2017) | USA | Case study | Neonates | 1 | **Y** | **Y** | **Y** | **N** | **N** | **N** | **Y** | **Y** |
| Kostogloudis (2015) | Greece | Case series | Neonates | 0 | **Y** | **Y** | **Y** | **N** | **N** | **N** | **Y** | **Y** |
| Launspach (2021) | Germany | Case study | Child | 1 | **Y** | **Y** | **Y** | **N** | **N** | **N** | **NA** | **Y** |
| Moon (2012) | Korea | Case series | Neonates | 0 | **Y** | **Y** | **Y** | **N** | **N** | **N** | **Y** | **N** |
| Murphy (2019) | Australia | Prospective cohort | Unknown | 0 | **Y** | **Y** | **Y** | **N** | **N** | **N** | **N** | **Y** |
| Odom (2018) | USA | Retrospective cohort | Mixed | 5 | **Y** | **Y** | **Y** | **N** | **N** | **N** | **N** | **Y** |
| Onesti (2012) | Italy | Case series | Neonates | 0 | **Y** | **Y** | **Y** | **N** | **N** | **N** | **Y** | **Y** |
| Park (2015) | Korea | Case study | Neonates | 1 | **Y** | **Y** | **Y** | **N** | **N** | **N** | **NA** | **Y** |
| Rodger (2018) | UK | Case study | Child | 1 | **Y** | **Y** | **Y** | **N** | **N** | **N** | **NA** | **Y** |
| Rueda-Mojica (2021) | Peru | Case study | Neonates | 1 | **Y** | **Y** | **Y** | **N** | **N** | **N** | **Y** | **Y** |
| Sivrioğlu (2014) | Turkey | Case series | Neonates | 0 | **Y** | **Y** | **Y** | **N** | **N** | **N** | **Y** | **Y** |
| Smaropoulos (2021) | USA | Case series | Neonates | 7 | **Y** | **Y** | **Y** | **N** | **N** | **N** | **Y** | **Y** |
| Sung (2016) | Korea | Case series | Neonates | 0 | **Y** | **Y** | **Y** | **N** | **N** | **N** | **Y** | **Y** |
| Talbot (2011) | USA | Case series | Neonates | 3 | **Y** | **Y** | **Y** | **N** | **N** | **N** | **NA** | **Y** |
| Ya-Min (2014) | Iran | Case series | Neonates | 0 | **Y** | **Y** | **Y** | **N** | **N** | **N** | **Y** | **Y** |
| Yan (2017) | China | Retrospective cohort | Mixed | 0 | **Y** | **Y** | **Y** | **N** | **N** | **N** | **Y** | **Y** |

^1^Number of cases extracted for case synthesis. ^2^Articles were assessed across four domains (Selection, Ascertainment, Causality, Reporting) and were graded “Higher Quality” if three or more domains were met.

Selection: Does the patient(s) represent(s) the whole experience of the investigator (center) or is the selection method unclear to the extent that other patients with similar presentation may not have been reported; Ascertainment: Was the exposure/outcome adequately ascertained; Causality: Were other alternative causes that may explain the observation ruled out/a challenge/rechallenge phenomenon/ dose–response effect/was follow-up long enough for outcomes to occur; Reporting: Is the case(s) described with sufficient details to allow other investigators to replicate the research or to allow practitioners make inferences related to their own practice (12).

Abbreviations: N = No, quality domain was not met; NA = no available extractable case data; Y = Yes, quality domain was met

Supplementary Table 1. Search strategy

| **PubMed Search Strategy** |
| --- |
| ("Extravasation of Diagnostic and Therapeutic Materials"/exp OR extravasation*:ti,ab OR extravasate*:ti,ab OR "Extravasation of Diagnostic and Therapeutic Materials/prevention and control"/exp OR "Extravasation of Diagnostic and Therapeutic Materials/etiology"/exp OR "Extravasation of Diagnostic and Therapeutic Materials/therapy"/exp) AND (paediatric*:ti,ab OR pediatric*:ti,ab OR child*:ti,ab OR infant*:ti,ab OR "young person":ti,ab OR neonat*:ti,ab OR neo-nat*:ti,ab OR newborn*:ti,ab OR new-born*:ti,ab OR toddler*:ti,ab OR Infant/exp OR Child/exp OR "young child*":ti,ab) AND (hyaluronidase:ti,ab OR hyaluroni*:ti,ab OR medic*:ti,ab OR saline:ti,ab OR salin*:ti,ab OR wash$out*:ti,ab OR suction-washing:ti,ab OR irrigation:ti,ab OR surgery:ti,ab OR surgical:ti,ab OR excision:ti,ab OR warm*:ti,ab OR cool*:ti,ab OR cold*:ti,ab OR heat*:ti,ab OR hot*:ti,ab OR "peripheral intravenous cannula*":ti,ab OR conservative*:ti,ab OR non-operative*:ti,ab OR non-surg*:ti,ab OR therapeut*:ti,ab OR manage*:ti,ab OR treatment*:ti,ab OR intervention*:ti,ab OR non$operative*:ti,ab OR non$surg*:ti,ab OR elevat*:ti,ab OR dress*:ti,ab OR surg*:ti,ab OR intervention*:ti,ab OR excis*:ti,ab OR infiltrat*:ti,ab OR manage*:ti,ab OR operat*:ti,ab OR bedside*:ti,ab OR liposuct*:ti,ab OR fasciotom*:ti,ab OR sub$cut*:ti,ab OR subcut*:ti,ab OR inject*:ti,ab OR emollient*:ti,ab OR antidote*:ti,ab OR sodium$thiosulfate*:ti,ab OR mechlorethamine*:ti,ab OR dexrazoxane*:ti,ab OR cortico$steroid*:ti,ab OR steroid*:ti,ab OR corticosteroid*:ti,ab OR topical*:ti,ab OR cream*:ti,ab OR ointment*:ti,ab OR ascorbic*:ti,ab OR vitamin$c*:ti,ab OR squeeze*:ti,ab OR tissu*:ti,ab OR prevent*:ti,ab OR treat*:ti,ab OR "therapy*":ti,ab) |
